# Physician-reported barriers to using evidence-based recommendations for low back pain in clinical practice: a systematic review and synthesis of qualitative studies using the Theoretical Domains Framework

**DOI:** 10.1186/s13012-019-0884-4

**Published:** 2019-05-07

**Authors:** Amanda M. Hall, Samantha R. Scurrey, Andrea E. Pike, Charlotte Albury, Helen L. Richmond, James Matthews, Elaine Toomey, Jill A. Hayden, Holly Etchegary

**Affiliations:** 1 Primary Healthcare Research Unit (PHRU), Faculty of Medicine, Memorial University, Room 417 | Janeway Hostel, Health Sciences Centre, 300 Prince Philip Parkway, St. John’s, NL A1B 3V6 Canada; 2 Nuffield Department of Primary Care Health Sciences, Medical Sciences Division, University of Oxford, Oxford, UK; 3 Centre for Rehabilitation Research in Oxford, University of Oxford, Oxford, UK; 4 School of Public Health, Physiotherapy and Sports Science, University College Dublin, Dublin, Ireland; 5 School of Psychology, National University of Ireland Galway, Galway, Ireland; 6 Department of Community Health & Epidemiology, Dalhousie University, Halifax, Nova Scotia Canada; 7 Clinical Epidemiology, Faculty of Medicine, Memorial University, St. John’s, Newfoundland and Labrador Canada

**Keywords:** Theoretical Domains Framework, Implementation, Behaviour change, Low back pain, Guidelines

## Abstract

**Background:**

Adoption of low back pain guidelines is a well-documented problem. Information to guide the development of behaviour change interventions is needed. The review is the first to synthesise the evidence regarding physicians’ barriers to providing evidence-based care for LBP using the Theoretical Domains Framework (TDF). Using the TDF allowed us to map specific physician-reported barriers to individual guideline recommendations. Therefore, the results can provide direction to future interventions to increase physician compliance with evidence-based care for LBP.

**Methods:**

We searched the literature for qualitative studies from inception to July 2018. Two authors independently screened titles, abstracts, and full texts for eligibility and extracted data on study characteristics, reporting quality, and methodological rigour. Guided by a TDF coding manual, two reviewers independently coded the individual study themes using NVivo. After coding, we assessed confidence in the findings using the GRADE-CERQual approach.

**Results:**

Fourteen studies (*n* = 318 physicians) from 9 countries reported barriers to adopting one of the 5 guideline-recommended behaviours regarding in-clinic diagnostic assessments (9 studies, *n* = 198), advice on activity (7 studies, *n* = 194), medication prescription (2 studies, *n* = 39), imaging referrals (11 studies, *n* = 270), and treatment/specialist referrals (8 studies, *n* = 193). *Imaging behaviour* is influenced by (1) *social influence*—*fr*om patients requesting an image or wanting a diagnosis (*n* = 252, 9 studies), (2) *beliefs about consequence—*physicians believe that providing a scan will reassure patients (*n* = 175, 6 studies), and (3) *environmental context and resources—*physicians report a lack of time to have a conversation with patients about diagnosis and why a scan is not needed (*n* = 179, 6 studies). *Referrals to conservative care* is influenced by *environmental context and resources*—long wait-times or a complete lack of access to adjunct services prevented physicians from referring to these services (*n* = 82, 5 studies).

**Conclusions:**

Physicians face numerous barriers to providing evidence-based LBP care which we have mapped onto 7 TDF domains. Two to five TDF domains are involved in determining physician behaviour, confirming the complexity of this problem. This is important as interventions often target a single domain where multiple domains are involved. Interventions designed to address all the domains involved while considering context-specific factors may prove most successful in increasing guideline adoption.

**Registration:**

PROSPERO 2017, CRD42017070703

**Electronic supplementary material:**

The online version of this article (10.1186/s13012-019-0884-4) contains supplementary material, which is available to authorized users.

## Background

Low back pain (LBP) is a common condition that has been reported by the Global Burden of Disease Study to cause more disability than any other condition [1]. International guidelines agree on best evidence-based care for managing back pain which includes a series of recommendations outlined in Table 1 [2]. This care begins with performing a diagnostic triage to rule out rare cases of specific spinal pathology or radicular syndrome. For non-specific cases, investigations are not recommended and management should include reassurance about good prognosis, advice to stay active and avoid bed rest, a short course of a simple pain medication, and self-care strategies. It is recommended to assess yellow flags in order to tailor education, reassurance, and advice. If patients have not improved after 6 weeks, referral to adjunct conservative management (exercise therapy, cognitive behaviour therapy (CBT), and pain management programs) is recommended. However, these recommendations are not routinely used by health professionals in primary care [3, 4]. Common problems include inappropriate advice regarding rest and activity, unnecessary referrals for imaging and surgery, and over-prescription of opioid medicines (e.g. codeine, oxycodone) [3–6]. The result of non-adherence to practice guidelines is poor health outcomes for patients and unnecessary costs and resource use for the health system [7, 8].

**Table 1:** Target clinical behaviours

Clinical behaviour	Description
All patients presenting with LBP	
1. Perform assessment and diagnostic triage	Assessed in-clinic by conducting a focused history and physical exam (including assessing for red flags (alerting features)) suggesting specific pathology, neurological tests for radicular syndromes, and assessment of yellow flags (presence of psychosocial risk factors). Then, exclude non-spinal pain causes (e.g. hip pathology, vascular causes); and provide a diagnosis of: specific pathology (e.g. fracture, infection, cauda equina), radicular syndrome (e.g. spinal stenosis or radiculopathy) or non-specific LBP (e.g. presumed lumbar musculoskeletal origin with no tests to specify pathoanatomical pain source)
For non-specific LBP	
2. Provide patient education	Provide advice on self-management strategies with education about their condition and the associated harms of bed rest and benefits of remaining active with staged resumption of normal activities where necessary.
3. Provide simple analgesics	Start with simple analgesics. Use non-steroidal anti-inflammatory medications for a short time after consideration of side effects and avoid opiates.
4. Only image in those with suspected spinal pathology	Imaging should only be used when a thorough patient history and physical exam indicate a serious specific cause for LBP. Do not order imaging for patients with non-specific LBP.
5. Referral to adjunct treatments or specialists	Referral to evidence-based adjunct conservative therapies such as physiotherapy for supervised exercise or pain management for more detailed education on pain management strategies and a goal-oriented plan of care. Referrals to specialists for surgical consultations should be reserved for those who continue to have radicular symptoms at 12 weeks and do not respond to conservative care, in which case surgery may be considered a possible treatment.

To increase uptake of guideline-based care, we need to develop effective interventions that will support health professionals to change their behaviour and adopt recommendations in their daily practice. Grol and Wensing’s model for developing behaviour change interventions dictates that in order to change behaviour, it is necessary to understand why the problem behaviour is occurring [9, 10]. Once the target population and target behaviour for change have been identified, the next step is to assess the barriers and enablers for performing the target behaviour and then select the appropriate behaviour change intervention strategies. This assessment and selection process should use a theoretical approach based on established psychological theories of behaviour change [9–12].

Michie et al. have developed a series of interacting frameworks including the Theoretical Domains Framework (TDF) and the Behaviour Change Techniques (BCT) Taxonomy [13–16] that identity factors influencing health professionals’ implementation of evidence-based guidelines into practice and appropriate interventions to address identified barriers. The TDF consists of 14 domains synthesised from 36 behaviour change theories and includes over 128 key theoretical constructs in a single framework [13, 15]. Examples of domains include *knowledge*, *skills*, *beliefs about capabilities*, *and social influences*. The BCT Taxonomy provides a list of 93 techniques that can be used to change behaviour such as *information about outcomes*, *modelling*, *rehearsal*, *monitoring*, *feedback*, and *credible source* [16]. Michie et al. also provided guidance on how to choose the most appropriate BCTs to address each of the 14 domains to achieve the most effective outcomes. Using this approach, we can identify which domains are relevant for adopting back pain management guidelines and then use the BCTs linked to those domains to develop appropriate interventions. This approach has been increasingly used to understand barriers and enablers to implementing or de-implementing guidelines for a variety of behaviours (e.g. adopting physical activity or weight management guidelines [17] or guidelines for reducing unnecessary preoperative testing.) [18].

A recent systematic review explored how multiple health professionals (e.g. physicians, physiotherapists, and chiropractors) use guidelines for managing LBP [19]. These results (including studies up to 2014) highlighted that barriers to implementing guidelines for back pain is a complex issue likely influenced by the patient. The results, however, do not provide specific information about the barriers physicians encounter when trying to (1) perform the recommended in-clinic diagnostic assessments, (2) avoid prescribing opioids, or (3) avoid referring for an image when it is not indicated. We will build on the work of Slade et al. by updating the systematic review in this area and analysing the data systematically and comprehensively using the TDF [14].

### Aim

This review synthesises the evidence from qualitative study designs regarding physicians’ barriers and enablers to providing evidence-based care for LBP in clinical practice settings. We used the TDF to organise the findings and map specific barriers and enablers to individual guideline recommendations. The results give direction to the design of interventions aimed at increasing physician compliance with providing evidence-based care for LBP.

## Methods

The protocol for this review was prospectively registered on PROSPERO (CRD42017070703; https://www.crd.york.ac.uk/prospero/display_record.php?RecordID=70703).

### Searches

An experienced librarian adapted the search strategy used by Slade et al. [19] which included papers up to 2014 to update the search for additional studies. She searched EMBASE and PubMed (Medline) for articles published between 2014 to June 2018 (Additional file 1). We also conducted forward and backward citation tracking for all included studies to identify any studies that might have been missed in the electronic search and contacted content experts or known researchers in this field.

### Study inclusion and exclusion criteria

All titles identified by the initial search were combined in Endnote and duplicates were removed. Article titles and abstracts of all studies identified in the search were initially screened by one reviewer (SS) using a screening template that included the pre-specified eligibility criteria. We included articles that (1) reported the results of original studies, (2) contained a qualitative method (e.g. focus group, interview), (3) included physicians as the study participants, (4) discussed the physician’s perspective of using guideline-based treatment recommendations for treating low back pain, and (5) included qualitative data on at least one of our outcomes of interest. These included assessment (e.g. diagnostic triage, red flag assessment, physical assessment), imaging tests (e.g. x-ray, MRI, CT scan), treatments (e.g. medication, advice), or referrals provided (e.g. specialist, physiotherapists, massage, chiropractor, multidisciplinary treatment). Two reviewers (SS, AH) screened the remaining titles and abstracts to identify studies requiring full-text review. Full-text review was completed by two reviewers (SS, AH) to select the final articles included in this review. If consensus could not be reached on whether or not an article should be included, a third reviewer (HR) was available to mediate disagreements; mediation was not necessary.

#### Study quality assessment (reporting and methodological rigour)

Two reviewers (AH, SS) independently assessed reporting quality using the Critical Appraisal and Skills Programme (CASP) in combination with the Consolidated criteria for reporting qualitative research (COREQ) 32-item checklist [20, 21]. The CASP checklist includes ten reporting categories (*aims*, *approach*, *design*, *recruitment*, *data collection*, *analysis*, *researcher-participant relationship*, *ethical issues*, *findings*, *and value*) to judge transparency of reporting and inform the assessment of rigour, credibility, and relevance of a study. The COREQ provides further guidance on the specific items to assess within each of the domains and is recommended by the Enhancing the QUAlity and Transparency Of health Research (EQUATOR) network. While there is a lack of consensus on how to judge methodological quality in qualitative research, four of the CASP domains pertaining directly to methodology provided the foundation on which we based our assessment of methodological rigour: (1) recruitment and selection methods, (2) data collection procedures, (3) researcher-participant relationship considerations, and (4) analysis methods. Judgements on each of the four domains were weighted equally to provide an overall score that determined if the study was ranked as having good, moderate, or low methodological rigour.

#### Data extraction strategy

All data extraction and assessments were carried out by two reviewers independently (AH, SS). Data was compared and discrepancies resolved via consensus. A data extraction template was used to collect the following information from each included paper: study aim and design, setting (rural or urban), participants (e.g. the physician’s area of practice such as family medicine, emergency medicine, etc.), sample size, sampling strategy, data collection, analytic approach, and main findings (including themes and sub-themes).

#### Data synthesis and presentation

##### Target behaviours

Five physician behaviours were defined a priori to focus the results. These were based on three of the latest international guidelines for physicians regarding how to manage low back pain [22–24]. The behaviours included (1) performing recommended diagnostic assessments (e.g. clinical history, red/yellow flags, physical/neurological testing), (2) providing recommended advice on activity, (3) prescribing recommended medications (i.e. simple analgesics or opioids), (4) not ordering imaging investigations unless required, and (5) providing referrals for recommended treatments (i.e. exercise therapy). Synthesis was conducted for each of the five behaviours separately.

##### TDF synthesis

Two researchers (AH, SS) independently coded the complete results section of the included studies using a framework synthesis approach; NVivo 11 software was used for data management. The framework was defined a priori to reflect the 14 domains in the TDF. Within each domain, there are several sub-domains that help to clarify the determinant of behaviour. A coding manual was developed by four authors (CA, HR, SS, AH) to operationalise the TDF for the context of this specific review and to help with coding consistency. The coding manual was reviewed with two additional authors who have health psychology backgrounds (HE, JM).

The first step involved independent coding by two reviewers of all data in the included studies according to the 14 TDF domains. This included coding data such as authors’ descriptions of the results and illustrative participant quotes provided in the results section (or results tables) of included studies. We compared independent coding and resolved discrepancies through discussion. When agreement could not be reached, a third assessor was consulted to mediate (either of CA, HE). Secondly, data were further coded according to the TDF sub-domains. For example, all data coded under the TDF domain “social influence” was further coded into one or more of the social influence sub-domains (e.g. social pressure or inter-group conflict, etc.). Once both reviewers had independently coded the data into TDF sub-domains, a summary of the coding results was reviewed with the team (including key informant physicians and health psychologists) for discussion and agreement on coding interpretations. Lastly, the themes at each sub-domain were organised into the corresponding behaviour category and a content analysis was undertaken which involved providing the number of contributing studies for each theme and describing the relevant study information to prepare the data for the confidence assessment using the Confidence in the Evidence from Reviews of Qualitative research (CERQual) approach [25].

##### Confidence in the findings for each of the five target behaviours

We used the CERQual approach developed by the Grading of Recommendations Assessment, Development, and Evaluation (GRADE) Working Group [25]. The GRADE-CERQual approach provides guidance for assessing how much confidence to place in review findings from qualitative evidence syntheses. In this review, review findings are the themes categorised at the TDF sub-domain level for each of the five target behaviours. For each target behaviour, all review findings were graded using the CERQual approach. This includes using a systematic and transparent framework for assessing confidence of the review finding based on consideration of four components: (1) methodological limitations, (2) coherence, (3) adequacy of data, and (4) relevance. There are four levels of confidence: high, moderate, low, and very low. The confidence level starts at high and is downgraded according to judgements based on the four components. In this review, the confidence level was not downgraded if all components were judged to have minor or very minor concerns. We downgraded the confidence in a review finding by one level for each of the four components that were judged to have moderate or serious concerns [25–31] (Additional file 2).

## Results

### Summary of included studies

**Fig. 1:**
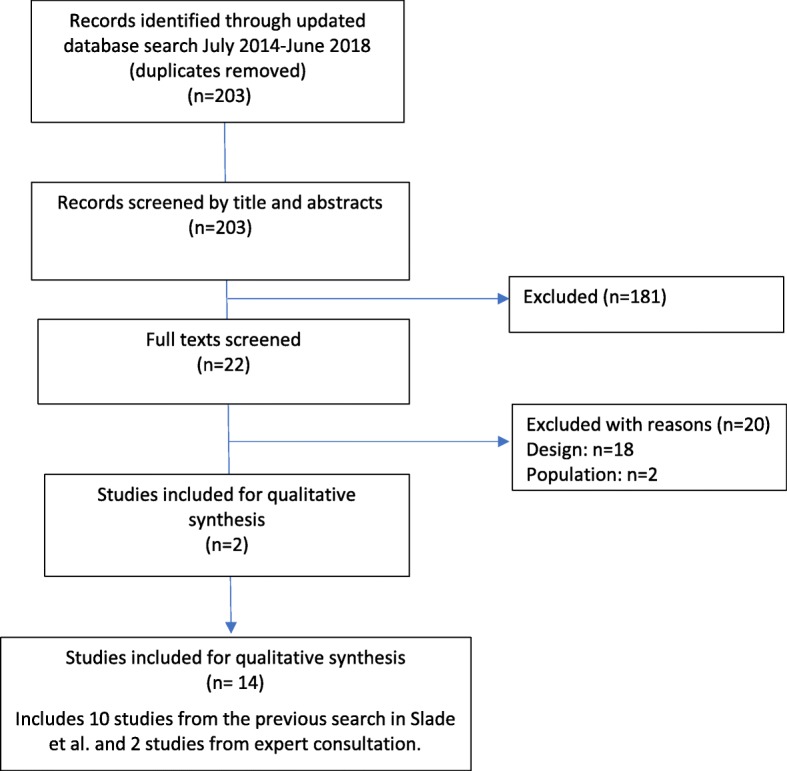
PRISMA flow diagram

In the updated search (January 2014–June 2018), we identified a total of 203 studies after duplicates were removed. We screened 22 full texts, of which 2 were eligible and identified a further 2 from grey literature, reference list searching, and consultation with experts. Combined with the 10 eligible studies from the initial search conducted in the Slade et al. review, we identified a total of 14 studies (including 318 participants) that assessed physicians’ perspectives of adhering to guideline-recommended behaviours for managing low back pain [32–45]. A description of the study identification and selection is outlined in the PRISMA flow diagram (Fig. 1). The studies were conducted between the years of 1998 and 2016 in both rural and urban settings in ten countries including three in the UK [32, 33, 35], two in Canada [42, 43], two in New Zealand [36, 38], and one each in Israel [37], Germany [34], Norway [39], the USA [45], Australia [40], the Netherlands [44], and Ireland [41]. Data were collected via semi-structured interviews (*n* = 8) or focus groups (*n* = 6), and the majority (*n* = 10) reported used a purposive sampling strategy (Table 2).

**Table 2:** Description of included studies

Study, year Country	GP (*n*)	Study aim	Data source	Recommendations discussed in thematic analysis					Method rigour
				Assessment & diagnosis (9)	Treatment referral (*n* = 8)	Medication (*n* = 2)	Activity advice (*n* = 7)	Imaging (*n* = 11)	
Bishop 2015 [32] UK	16	To clarify the decision-making processes re: particular treatments to LBP patients	Interview	✓	✓				Moderate
Breen 2007 [33] UK	21	To examine GP attitudes to managing acute LBP as a biopsychosocial problem	Focus group (*n* = 3)	✓	✓		✓	✓	Moderate
Chenot 2008 [34] GER	72	To explore the acceptance of GL content and perceived barriers to implementation	Focus group		✓		✓	✓	Low
Corbett 2009 [35] UK	10	To explore the attitudes and self-reported behaviour of GPs in relation LBP GL	Interview	✓			✓	✓	Good
Crawford 2007 [36] NZ	11	To understand GPs experience in identifying and managing *Yellow Flags*	Interview	✓					Moderate
Dahan 2007 [37] IS	38	To identify barriers and facilitators that GPs experience when using LBP GL	Focus group (*n* = 4)	✓				✓	Moderate
Darlow 2014 [38] NZ	11	To explore GPs’ underlying beliefs about LBP and how these beliefs influence their management	Interview	✓			✓		Good
Espeland 2003 [39] NO	13	To understand GPs barriers to using GL and what they think affects their ordering x-rays	Focus group (*n* = 3)					✓	Good
French 2012 [40] AU	42	To identify the barriers and enablers to restricting use of x-rays and providing advice on remaining active	Focus group	✓			✓	✓	Good
Fullen 2008 [41] IRE	7	To understand factors that impact on GPs management of chronic LBP	Interview		✓		✓	✓	Good
Green 2015 [42] CA	10	To understand the factors that influence ordering MRI for without “red flags.”	Interview		✓			✓	Moderate
Poitras 2012 [43] CA	8	To evaluate barriers for using GL for preventing LBP disability	Interview	✓	✓	✓		✓	Good
Schers 2001 [44] NL	31	To explore factors that determine nonadherence to the LBP GL	Interview	✓	✓	✓	✓	✓	Moderate
Shye 1998 [45] USA	28	To understand nonadherence to imaging GL for LBP	Focus group (*n* = 4)		✓			✓	Moderate

### Study quality of reporting and methodological rigour

**Fig. 2:**
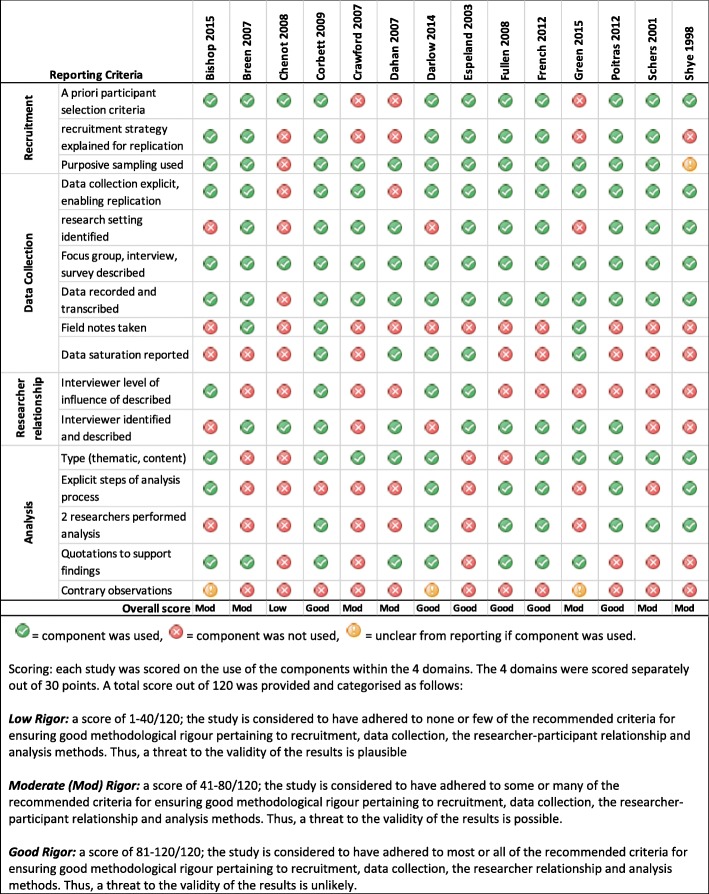
Methodological rigour assessment

Overall, most studies provided sufficient information on the aim, approach, and design. While most studies reported using inclusion criteria, only half of the studies described the criteria in sufficient detail for replication. Important areas that were poorly reported (i.e. less than half of the studies reported on the area) included information about whether a theoretical framework was used, if data saturation was achieved, interviewer influence, ethical approval, and the steps of the analysis process (i.e. the number of reviewers and inclusion of quotations to support findings). A full description of the reporting assessment can be found in Additional file 3. Using the four CASP domains pertaining to methodology, six studies were judged to have good methodological rigour [35, 38–41, 43], seven had moderate methodological rigour [32, 33, 36, 37, 42, 44, 45], and one had low methodological rigour [34] (Fig. 2).

### Synthesis

**Fig. 3:**
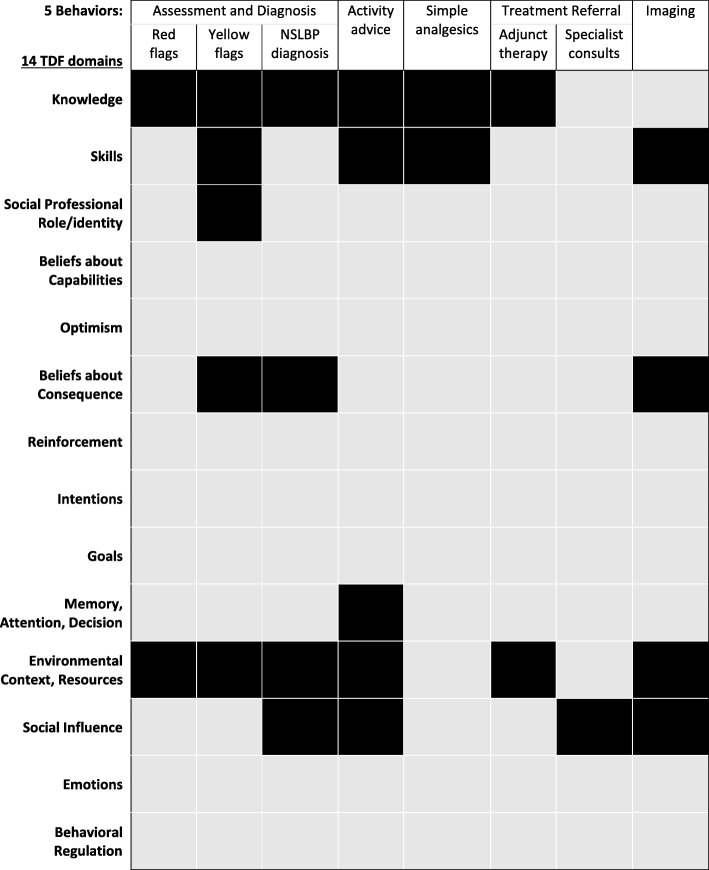
Summary of TDF domains identified for each behaviour. Legend: Grey box indicates no themes were identifed at this domain. Black box indicates that theme(s) were identified at this domain

Most of the 14 included studies assessed the physicians’ perspective of adhering to more than one of the five target behaviours. These included recommendations pertaining to in-clinic diagnostic assessments and providing a diagnosis of non-specific low back pain (*n* = 9), providing advice on activity and rest (*n* = 7), imaging investigations (*n* = 11), medication (*n* = 2), and referrals to treatment providers (*n* = 8). Figure 3 outlines the major TDF domains identified for each of the five target behaviours. A summary of our confidence in the findings for each of the five target behaviours listed in Table 1 is provided in Tables 3, 4, 5, 6, and 7. For three of the five behaviours (i.e. assessments, activity advice, and medication), the identified themes were not supported by enough contributing studies and/or the contributing studies were judged to have serious methodological limitations. In these cases, the level of confidence was downgraded to a moderate or low level of confidence based on data quantity (e.g. few contributing studies) and methodological rigour (e.g. severe methodological limitations). In the tables, we report all themes for each behaviour and the level of confidence for each theme. In the text below, we focus our reporting to themes with high confidence only.

**Table 3:** Summary of findings regarding physician-reported barriers for performing recommended assessments and diagnosis

TDF domain	TDF sub-domain	Specific theme from the study	Studies (participants)	Confidence in the evidence	Explanation
Performing all assessments					
Environment context and resources	Resources	GP’s do not have enough time to complete all assessments, full history, full exam and full neurological assessment “you are lucky to have a 10-minute interview, consultation, to actually obtain a full history, and full examination, full back neurological assessment is hard”	3 (42)	Moderate^3^	No or very minor concerns regarding methodology, coherence, and relevance. Moderate or serious concerns about adequacy
Assessing for red flags					
Knowledge	Scientific knowledge	Lack of awareness of red flags for serious pathology “low awareness of LBP red flags and skills in how to identify them”	1 (42)	Low^2–3^	No or very minor concerns regarding methodology and relevance. Moderate or serious concerns regarding coherence and adequacy
Assessing for yellow flags					
Knowledge	Scientific knowledge	A general lack of knowledge regarding what yellow flags were or their importance in relation to the management of low back pain There were a range of views regarding when patient attitudes and beliefs become important, reflecting general uncertainty about how and why they influence pain and outcomes	4 (50)	Moderate^3^	No or very minor concerns regarding methodological limitations coherence, or relevance. Moderate or serious concerns regarding adequacy
Social/professional role and identity	Professional role	GP’s do not believe it is their role to assess psychosocial factors “All but 1 GP…thought that the assessment of psychosocial factors was not their role”	2 (19)	Moderate^3^	No or very minor concerns regarding methodology coherence, or relevance. Moderate or serious concerns regarding adequacy
Beliefs about consequences	Beliefs	GP’s were reluctant to assess yellow flags because they were unsure that that managing yellow flags was a good idea as it may lead to conflict with the patient’s expectations of GP management and adversely affect the doctor-patient relationship. “…identifying and managing yellow flags could present conflicts with the patient’s expectations. They thought most patients expected to be managed using a biomedical and not a biopsychosocial approach, and the one found in the guidelines.”	3 (30)	Low^2–3^	No or very minor concerns regarding methodology and relevance. Moderate or serious concerns regarding coherence and adequacy
Environmental context and resources	Resources	Lack of time to assess this after all the other assessments “Most GPs mentioned that short treatment sessions, limited frequency and long intervals…restricted the capacity to assess and manage yellow flags.”	2 (19)	Moderate^3^	No or very minor concerns regarding methodology, coherence, and relevance. Moderate or serious concerns regarding adequacy
Skills	Skills	A lack of skills in how to assess yellow flags and facilitating discussion around their link to pain and recovery “The assessment of disability prognosis and psychosocial factors, essentially with questionnaires, was new for all GPs”	2 (19)	Moderate^3^	No or very minor concerns regarding methodology, coherence, and relevance. Moderate or serious concerns regarding adequacy
Providing a diagnosis of non-specific low back pain					
Knowledge	Scientific knowledge	Physicians thought they did not have sufficient understanding of anatomy to explain the natural healing process with non-specific low back pain. “GPs admitted difficulties in conveying the epidemiologic concept of unspecified LBP”	2(19)	Moderate^3^	No or very minor concerns regarding methodology, coherence, and relevance. Moderate or serious concerns regarding adequacy
Social influence	Social pressure	Patients want a “specific” diagnosis and lack of a “precise” diagnosis is not reassuring to them. “The problem with back pain is making a precise diagnosis. They always complain, ‘So what is the diagnosis?’…why do they want a CT? Simply in order to get a diagnosis.”	3 (80)	Moderate^3^	No or very minor concerns regarding methodology, coherence, and relevance. Moderate or serious concerns regarding adequacy
Beliefs about consequence	Outcome expectancy	Physicians did not believe providing a diagnosis of non-specific low back pain would help their patients recover because it is hard to understand. “I do not know what you are talking about so I am sure the patients would not. Non- specific, I mean it’s not really very helpful. they have either got muscle and joint and ligament pain or they have got nerve entrapment and that’s what they want to hear, they do not want to hear terms like non-specific back pain, they want to know what it is and what it is not.”	1 (16)	Very low^5^	Moderate or serious methodological concerns, coherence, and adequacy

CERQual Assessment: Confidence was downgraded 1 level for each of the four CERQual domains that had moderate or serious concerns defined as ^1^methodological limitation (the majority of the supporting data comes from studies with low methodological rigour threating the validity or reliability of the theme), ^2^coherence (the supporting data for the theme is drawn from studies that provided ambiguous or incomplete data that threatened the coherence of this theme), ^3^adequacy (the majority of the supporting data for the theme is drawn from few and/or small studies and the quality is superficial lacking sufficient richness to fully explore the theme), and ^4^relevance (the majority of the supporting data is of indirect, partial or unclear relevance to the theme. ^5^When the data come from a single study with few participants and of moderate rigour we downgraded to very low confidence. Please see Additional file 2 for a full description of the criteria used for assessing confidence in the evidence supporting the review findings using the CERQual approach

**Table 4:** Summary of findings regarding physician-reported barriers to providing activity advice

TDF domain	TDF sub-domain	Specific theme from the study	Studies (participants)	Confidence in the evidence	Explanation
Knowledge	Knowledge (scientific rationale)	Unsure about how, why and when exercise might be helpful “Views about activity were informed by guideline recommendations, but there was uncertainty as to how or why exercise might be helpful”	4 (114)	Moderate^3^	No or very minor concerns regarding methodology, coherence, and relevance. Moderate or serious concerns regarding adequacy.
	Procedural	Knowledge of what activity to advise on based on patient factors/circumstances “Much of the advice which participants reported conveying to patients contained mixed messages and reinforced the need to be active and protective at the same time.”	2 (21)	Moderate^3^	No or very minor concerns regarding methodology, coherence, and relevance. Moderate or serious concerns regarding adequacy.
Social influence	Intergroup conflict	Conflict between patient and physician wishes in which the physician felt the patient perceived physical activity to be counter intuitive and considered rest to be the best option or perception that patients did not want activity advice. “Changing the belief of patients who considered rest to be the best treatment could be challenging”	5 (131)	Moderate^3^	No or very minor concerns regarding methodology, coherence, and relevance. Moderate or serious concerns regarding adequacy.
Skills	Skills	Lack of skills to negotiate why activity is ok when the patient considered rest to be the best treatment “GPs reported that they felt patients perceived physical activity as counter-intuitive to the ‘warning sign’ that pain signified stress to the body, and therefore, one needed to rest.”	2 (21)	Moderate^3^	No or very minor concerns regarding methodology, coherence, and relevance. Moderate or serious concerns regarding adequacy.
Environment context and resources	Resources	Lack of time to give advice “Limited time to explain why patient does not need an x-ray and explain advice to stay active”	1 (42)	Low ^2,3^	No or very minor concerns regarding methodology and relevance. Moderate or serious concerns regarding coherence and adequacy.
Memory	Memory	Forget to give advice “GPs forget to give advice to stay active in standard consultation”	1 (42)	Low ^2,3^	No or very minor concerns regarding methodology and relevance. Moderate or serious concerns regarding coherence and adequacy.

CERQual Assessment: Confidence was downgraded 1 level for each of the four CERQual domains that had moderate or serious concerns defined as ^1^methodological limitation (the majority of the supporting data comes from studies with low methodological rigour threating the validity or reliability of the theme), ^2^coherence (the supporting data for the theme is drawn from studies that provided ambiguous or incomplete data that threatened the coherence of this theme), ^3^adequacy (the majority of the supporting data for the theme is drawn from few and/or small studies and the quality is superficial lacking sufficient richness to fully explore the theme), and ^4^relevance (the majority of the supporting data is of indirect, partial or unclear relevance to the theme. ^5^When the data come from a single study with few participants and of moderate rigour we downgraded to very low confidence. Please see Additional file 2 for a full description of the criteria used for assessing confidence in the evidence supporting the review findings using the CERQual approach

**Table 5:** Summary of findings regarding physician-reported barriers to prescribing simple analgesics instead of stronger medication

TDF domain	TDF sub-domain	Specific theme from the study	Studies (participants)	Confidence in the evidence	Explanation
Knowledge	Knowledge of condition/scientific rationale	Disagreement with guideline advice regarding simple analgesics, muscle relaxants and opioids. “Also, most GPs disagreed with the guidelines on opioid use, stating that these were often necessary to effectively manage pain despite the associated adverse effects.”	2 (39)	Moderate^3^	No or very minor concerns regarding methodological limitations, coherence and relevance Moderate or serious concerns regarding adequacy
Skills	Skills	Perception that patients want something stronger and that it is difficult to “sell” simple analgesics instead. “Most GPs agreed with the guidelines advice to prescribe simple analgesics, and not a muscle relaxer. However, most said that they did not always adhere to this advice. Motives were diverse. Some could not sell “simple” analgesics to their patients…”	1 (31)	Very low^5^	Moderate or serious concerns regarding methodological limitations, coherence, and adequacy

CERQual Assessment: Confidence was downgraded 1 level for each of the four CERQual domains that had moderate or serious concerns defined as ^1^methodological limitation (the majority of the supporting data comes from studies with low methodological rigour threating the validity or reliability of the theme), ^2^coherence (the supporting data for the theme is drawn from studies that provided ambiguous or incomplete data that threatened the coherence of this theme), ^3^adequacy (the majority of the supporting data for the theme is drawn from few and/or small studies and the quality is superficial lacking sufficient richness to fully explore the theme), and ^4^relevance (the majority of the supporting data is of indirect, partial or unclear relevance to the theme. ^5^When the data come from a single study with few participants and of moderate rigour we downgraded to very low confidence. Please see Additional file 2 for a full description of the criteria used for assessing confidence in the evidence supporting the review findings using the CERQual approach

**Table 6:** Summary of findings regarding physician-reported perspective about why they use imaging to manage back pain

TDF domains	TDF sub-domain	Specific theme from the study	Studies (participants)	Confidence in the evidence	Explanation
Social influence	Social pressure	The patients ask for an image (in some cases because they want a diagnosis) and the GP feels pressured to request one. “A reason mentioned in all focus groups … was that patients with low back pain often expected, and sometimes even requested or demanded, these tests, despite the physician’s explanation that an imaging test was not (yet) warranted.”	9 (252)	High	No or very minor concerns regarding methodology, coherence, adequacy and relevance.
	Intergroup conflict	GPs will order an image to avoid conflict with a patient’s wishes. “GPs might order ‘non indicated’ X-rays …to limit conflict.”	3 (104)	Very Low ^1,2,3^	No or very minor concerns regarding relevance. Moderate or serious concerns regarding methodology, coherence, and adequacy.
Beliefs about consequence	Consequences	GPs fear blame or legal action if they do not send for scans. “GPs said they ordered radiography because of…. possible legal actions.”	4 (126)	Low ^2,3^	No or very minor concerns regarding methodology and relevance. Moderate or serious concerns regarding coherence and adequacy.
	Consequences	GPs may order an image if they thought it would improve trust in the doctor-patient relationship. “If they thought that ordering an imaging test would enhance patient’s trust (or that denying one might undermine it), a test might be ordered when it was not strictly medically indicated”	5 (101)	Low ^2,3^	No or very minor concerns regarding methodology and relevance. Moderate or serious concerns regarding coherence and adequacy.
	Outcome expectancy	GPs believe scans will reassure patients that nothing is wrong. “Sometimes an x-ray can take away the fear, and thus prevent chronicity.’ Another agreed. ‘When patients worry, that is a heavy argument; you need the reassurance (gained from further tests) to go on with the patient”	6 (175)	High	No or very minor concerns regarding methodology, coherence, adequacy and relevance.
Skills	Skills	Lack of communication skills to convince the patient that there was nothing wrong. “If it seemed unlikely they would not be able to convince the patient with a reasonable effort, they would simply order the test”	3 (101)	Moderate ^3^	No or very minor concerns regarding methodology, coherence and relevance Moderate or serious concerns regarding adequacy.
	Skills	GPs thought they used radiography because they lacked skills in clinical examination of the back. “Some GPs thought they overused radiography because they lacked skills in clinical examination: We have got so much to work with that…many (of us)…will never be any good at examining a back.”	1 (13)	Low ^2,3^	No or very minor concerns regarding methodology and relevance. Moderate or serious concerns regarding coherence and adequacy.
Environment context and resources	Resources	GP’s do not have enough time to negotiate or explain the diagnosis so they order an x-ray. “Sometime I find myself referring a patient for X-ray in order to clear the waiting room and allow myself two minutes of breathing time. Meanwhile the patient keeps quiet, while I write the referral. Sometimes you find yourself doing this and it goes against any reasoning or logic”	6 (179)	High	No or very minor concerns regarding methodology, coherence, adequacy, and relevance.
	Resources	If GPs perceive a long wait for an image, and they may eventually want to order one, they may order it early, even if not indicated at that time. “They indicated if they perceive there was a long waiting period for a service the patient might eventually need (such as a CT, or MRI scan), they might order one earlier than they thought was really necessary just to get the patient in the queue.”	2 (38)	Low^2,3^	No or very minor concerns regarding methodology and relevance. Moderate or serious concerns regarding coherence and adequacy.
	Resources	There is no alternative to offer the patient instead of the image.	2 (?)	Moderate ^3^	No or very minor concerns regarding methodology and relevance. Moderate or serious concerns regarding coherence and adequacy.
	Organisational culture	GPs would refer if the patient may need them for medico-legal cases, e.g. if the patient needed to make an insurance claim later on. “GPs also ordered radiography to secure documentation in case the patient claimed for insurance compensation…”	4 (72)	Moderate ^3^	No or very minor concerns regarding methodology, coherence and relevance Moderate or serious concerns regarding adequacy.
	Organisational culture	GPs refer for an image if other treatment providers (physiotherapists, specialists) required a scan before evaluating the patient. “GPs said physiotherapists might want radiography before giving (further) treatment, surgeons before evaluating patients clinically, and radiologists before or in addition to performing CT.”	4 (93)	Moderate ^3^	No or very minor concerns regarding methodology, coherence and relevance Moderate or serious concerns regarding adequacy.
	Organisational culture	GPs reported sending for scans if they were required for sick certification or short-term disability. “Social security might request radiography to establish facts before considering (continued) sickness certification or disability pension (…contributing to and endless dance in the X-ray corridors). To help patients get further economical support, GPs usually complied with such pressures, although they often found radiography unnecessary by clinical criteria.”	2 (23)	Moderate ^3^	No or very minor concerns regarding methodology, coherence and relevance. Moderate or serious concerns regarding adequacy.

CERQual Assessment: Confidence was downgraded 1 level for each of the four CERQual domains that had moderate or serious concerns defined as ^1^methodological limitation (the majority of the supporting data comes from studies with low methodological rigour threating the validity or reliability of the theme), ^2^coherence (the supporting data for the theme is drawn from studies that provided ambiguous or incomplete data that threatened the coherence of this theme), ^3^adequacy (the majority of the supporting data for the theme is drawn from few and/or small studies and the quality is superficial lacking sufficient richness to fully explore the theme), and ^4^relevance (the majority of the supporting data is of indirect, partial or unclear relevance to the theme. ^5^When the data come from a single study with few participants and of moderate rigour we downgraded to very low confidence. Please see Additional file 2 for a full description of the criteria used for assessing confidence in the evidence supporting the review findings using the CERQual approach

**Table 7:** Summary of findings regarding physician-reported barriers to referring for recommended conservative or specialist consultations

TDF Domain	TDF Sub Domain	Specific theme from the study	Studies (participants)	Confidence in the evidence	Explanation
Behaviour: referring to adjunct conservative treatments: physiotherapy or pain management programs					
Knowledge	Knowledge of condition/scientific rationale	GPs unfamiliar with conservative interventions besides medication such as CBT “Most GPs were unfamiliar with the conservative interventions other than medication, such as cognitive-behavioural therapy, spinal manipulations, and exercises.”	2 (80)	Very low ^1,3^	Moderate or serious concerns regarding methodology, coherence and adequacy.
	Scientific rationale	Do not believe that referrals to physical therapy work “It was striking that half of the GPs did not consider physical therapy to be beneficial at all. One said, ‘I think physical therapy is never necessary for this matter.’”	1 (31)	Very low ^5^	Moderate or serious concerns regarding, coherence and adequacy.
Environment context and resources	Resources	Lack of services and long wait times for physiotherapy “…structural barriers like lack of access to recommend treatment options prevent guideline-concordant patient management”	5 (82)	High	No or minor concerns regarding methodology, coherence, adequacy, and relevance.
Behaviour: referring to specialist services: orthopaedics; surgical consults					
Social influence	Social pressure	Physicians are often pressured to make referrals even if they do not think they are required because solicitors request then for medico-legal patients “Most of these medico-legal patients are referred to us by their solicitors for referral to orthopaedics, I would often tell them to ask their solicitor to do the referral”	1 (7)	Low^2,3^	Moderate or serious concerns regarding methodology, coherence and adequacy.

CERQual Assessment: Confidence was downgraded 1 level for each of the four CERQual domains that had moderate or serious concerns defined as ^1^methodological limitation (the majority of the supporting data comes from studies with low methodological rigour threating the validity or reliability of the theme), ^2^coherence (the supporting data for the theme is drawn from studies that provided ambiguous or incomplete data that threatened the coherence of this theme), ^3^adequacy (the majority of the supporting data for the theme is drawn from few and/or small studies and the quality is superficial lacking sufficient richness to fully explore the theme), and ^4^relevance (the majority of the supporting data is of indirect, partial or unclear relevance to the theme). ^5^When the data come from a single study with few participants and of moderate rigour we downgraded to very low confidence. Please see Additional file 2 for a full description of the criteria used for assessing confidence in the evidence supporting the review findings using the CERQual approach

### Behaviour 1: use diagnostic triage/in-clinic assessment procedures (9 studies, *n* = 198)

Nine studies [32, 33, 35–38, 40, 43, 44] assessed and reported information on physicians’ perspectives using in-clinic diagnostic assessments and providing the patient with a diagnosis. These studies were conducted in six countries and used either focus groups (*n* = 3) and or semi-structured interviews (*n* = 6). It was challenging to assess barriers for this recommended behaviour from the guidelines because it includes multiple components (e.g. multiple in-clinic assessments and providing a diagnosis as described in Table 1). None of the studies we included in our review reported on all of the components included in this recommended behaviour; rather, they discussed the challenges with performing specific components. Five studies assessed the challenges of providing a diagnosis for non-specific low back pain, four examined using the yellow flag assessment, one examined using the red flag assessment, and three looked at performing all of the recommended assessments as a whole. The barriers identified for performing the different assessment types or providing a diagnosis were identified and are presented in Table 3. Of the ten themes identified for this behaviour, seven of the ten identified themes achieved a moderate level of confidence, two had a low level confidence and one had a very low level of confidence.

### Behaviour 2: provide activity advice (7 studies, *n* = 194)

Seven studies [33–35, 38, 40, 41, 44] assessed and reported information on physicians’ perspectives of providing advice on activity and/or rest to patients. These were conducted in six countries and used either focus groups (*n* = 3) and or semi-structured interviews (*n* = 4). Meta-synthesis identified six themes relating to five TDF domains (6 sub-domains) that reflected the main barriers and enablers for either not providing advice on activity and in some cases advising rest instead (Table 4). None of the identified themes achieved a high level of confidence to adequately explain barriers or enablers of this behaviour. Of the six themes, four were judged to have a moderate level of confidence and two achieved only a low level of confidence.

### Behaviour 3: prescribe simple analgesics for pain relief (2 studies, *n* = 39)

Two studies [43, 44] assessed and reported information on physicians’ perspectives of prescribing simple analgesics rather than muscle relaxants or opioids. These were conducted in two countries; one used a focus group and the other used semi-structured interviews. Meta-synthesis identified two themes relating to two TDF domains (2 sub-domains) that reflected why physicians would prescribe medications other than those recommended by the guidelines (Table 5). We do not have a high level of confidence that the identified themes adequately explain barriers or enablers of this behaviour. Of the two themes, one was judged to have a moderate level of confidence and one to have a very low level of confidence.

### Behaviour 4: do not refer for imaging unless red-flag indicated (11 studies, *n* = 270)

Eleven studies [34, 35, 37–45] assessed and reported information on physicians’ perspectives of using imaging. These were conducted in ten countries and used either focus groups (*n* = 6) and or semi-structured interviews (*n* = 5). Meta-synthesis identified 13 themes relating to four TDF domains (7 sub-domains) that reflected the main determinants for referring patients for imaging in the absence of red flags (Table 6). We have a high level of confidence that this behaviour is influenced by factors related to three themes: (1) *Social influence in the form of social pressure* from patients either requesting an image or wanting a diagnosis (*n* = 252, 9 studies) (2) *Beliefs about consequence* in that physicians believe that providing a scan will reassure patients that nothing is wrong (*n* = 175, 6 studies), and (3) *Environmental context and resources* where physicians report a general lack of time to have a full conversation with patients about diagnosis and why a scan is not needed (*n* = 179, 6 studies). Among the remaining ten themes, five achieved a moderate level of confidence, four a low level of confidence, and one a very low level of confidence.

### Behaviour 5: refer for other treatments (8 studies, *n* = 193)

Eight studies [32–34, 41–45] assessed and reported information on physician’s perspectives of referring patients for adjunct treatments such as physiotherapy, chiropractic, cognitive behavioural treatment, or pain management. These studies included a total of 193 physicians across six countries using either focus groups (*n* = 3) or semi-structured interviews (*n* = 5) for data collection. Meta-synthesis identified three themes relating to two TDF domains (3 sub-domains) that reflected physicians’ reasons for failing to refer patients to recommended adjunct conservative treatments (Table 7). We have a high level of confidence that this behaviour is influenced by environmental context and a lack of resources (*n* = 82, 5 studies). Physicians reported that long wait times or a complete lack of access to adjunct services prevented them from referring to services such as physiotherapy or pain management programs. The remaining two themes were judged to have a very low level of confidence.

## Discussion

### Summary

Searching the evidence up to July 2018, we found 14 studies of moderate or high methodological rigour that assessed barriers to the five main behaviours outlined in the guidelines. This review adds 3 new studies since the last thematic synthesis by Slade et al. [19] and provides the first theoretically driven synthesis to map specific barriers and enablers to individual guideline recommendations. In addition, we used the CERQual approach [25] to rigorously assess our confidence that the identified themes reliably explain the reasons for performing the behaviour. Taken together, this synthesis improves opportunities for evidence-informed behaviour change interventions.

Our systematic review found that physicians face barriers to providing evidence-based care for LBP that fall into seven of 14 TDF domains. The fact that between two to five TDF domains are involved in determining each of the five physician behaviours examined confirms the complexity of implementing guideline-based care for LBP. This issue is not unique to back pain; it is common to many health contexts (e.g. osteoarthritis, nutrition, physical activity, anti-psychotics, oral health, and weight management). For example, we identified several qualitative reviews investigating barriers to implementing guidelines that identified between 3 to 9 TDF domains as determinants of the target behaviour [46–51]. Further complicating matters, we found that different combinations of domains were implicated for each of the five behaviours. For example, the domains of knowledge, beliefs about capabilities, and social professional role/identify were identified as barriers to the recommended yellow flag assessment, but these domains were not identified as barriers to evidence-based referral for an X-ray. This is extremely important as interventions often target a single domain (e.g. knowledge) for a behaviour when in fact multiple domains may be implicated [52–58]. Additionally, an intervention based on a single domain may be used to try to change multiple behaviours. For example, using a knowledge-based intervention to change both the use of yellow flags and referrals for imaging will likely only be effective for using yellow flags, because knowledge is not a barrier for imaging.

### Previous implementation approaches

Many interventions have been developed to improve the adoption of LBP guidelines, most of which have focused on reducing imaging use (one of the more well-documented problem behaviours) [59, 60]. At least 17 interventions targeting this behaviour have been reported in the literature [52–58, 61–69]. Only six interventions focused on some of the barriers we identified [55, 57, 61–64]. None of the interventions included strategies to target the beliefs about consequence (e.g. physicians’ belief that a scan will reassure patients that nothing is wrong). Several targeted barriers related to resources (e.g. not having time to adequately explain the diagnosis), which included referrals to another health professional or service for assessment and diagnosis. Others targeted social pressure from patients who seek an image by providing physicians with additional communication skills to explain to the patient why an image is not needed. However, with the exception of one study [57], none of these interventions showed significant changes in image-ordering behaviour. Thus, to date, it appears that none of the approaches used have included strategies to address all three of the major barriers to not ordering imaging for low back pain.

### Theoretically-informed solutions to implementing LBP guidelines

Our theoretical analysis using the TDF provides a behavioural diagnosis of what specific barriers need to be addressed in order for each of target behaviours outlined in the low back pain guidelines to occur. This method is important because barriers identified using the TDF can be linked to appropriate intervention strategies using guidance from the BCT Taxonomy [15, 16]. For example, our results highlight that there are at least three main determinants of ordering imaging for low back pain relating to the TDF domains of (1) social influences, (2) beliefs about consequences, and (3) environmental context and resources. These barriers were common across studies irrespective of country, health system context, or data collection method. Thus, interventions aiming to change this behaviour should at least include behaviour change techniques that have been linked to these three TDF domains [16]. Examples of these techniques include (1) modelling/demonstration of the behaviour or social support; (2) information about the behaviour, self-monitoring, or feedback; and (3) adding objects to the environment or restructuring the physical environment, respectively. We provide an example of how these strategies could be used to build intervention components in Table 8. It is important to note that the TDF is just one framework that can be used to design behaviour-change interventions. Other psychological theories such as social cognitive theory [70], theory of planned behaviour [71], and the fear avoidance model [72] have been used to inform behaviour-change interventions. Regardless of which theory or theoretical framework is used, it is important to explicitly state how the theory is being applied to the intervention design by mapping the intervention components to the theoretical barrier it is aiming to target [73].

**Table 8:** Example of behaviour change techniques that could be combined to form a multifaceted intervention to target the 3 identified TDF barriers with a high level of confidence related to imaging

TDF domain	Specific theme from the study	One of the potential strategies (linked to TDF domains by the BCT taxonomy)
1. Social influence (Social pressure) and	The patient asks for an image (in some cases because they want a diagnosis) and the GP feels pressured to request one.	BCT: 6.1 Modelling or demonstrating the behaviour Example: Provide a video* or similar method that demonstrates their peers (respected members of their peer group) dealing with this situation; i.e. having a conversation with a patient about not ordering the image.
2. Beliefs about consequence (Outcome expectancy) and	GPs believe scans will reassure patients that nothing is wrong.	BCT: 5.1 provision of information about health consequences Example: provide information* about: • the negative consequences of ordering an image (i.e. delayed recovery, exposure to radiation, incidental findings, additional healthcare tests) • the comparative effectiveness of imaging on patient reassurance and recovery compared to other strategies
3. Environment context and resources Resources	GP’s do not have enough time to negotiate or explain the diagnosis so they order an x-ray.	BCT: 12.5 adding objects to the environment Example: provide an evidence-based leaflet and/or prescription pad* that provides information on back pain, diagnosis, prognosis, need for tests and self-management strategies specific to the patient.

*It would be important to test any information developed or provided as part of a BCT intervention to use to ensure it does indeed have the desired effect at the domain level. For example, any information developed for the patient should be tested with the patient to ensure it is understood by the patient

#### Limitations

While the studies included in this review were of predominantly good methodological rigour, we noted several limitations. First, important details regarding the sampling strategy were often missing. For example, purposive sampling was reported in most studies but little detail was provided on how this was achieved, thereby limiting our confidence in the representativeness of the data. Second, several studies described the behaviour of performing in-clinic assessments differently, with many reporting only a single aspect of this behaviour (e.g. only discussing yellow flags or red flag assessments). This limits our ability to make any firm conclusions about the barriers for behaviour as a whole due to insufficient information on all aspects. Lastly, while we consider the use of the TDF to categorise barriers a primary strength, it was sometimes challenging to categorise identified themes into only one TDF domain due to a lack of contextual information. To mitigate this issue, we employed a coding rule to report the TDF domain that best captured the main reason the behaviour was not performed.

### Strengths

The target population of this review was restricted to physicians only to ensure more relevant and coherent data on the barriers they face. A primary strength of this review is the utilisation of a theoretical framework to categorise the factors that influence the implementation of LBP guidelines. This allows for a deeper and more detailed understanding of the factors that influence each of the behaviours. We analysed the data separately for the five behaviours outlined in practice guidelines relevant to physicians, which aimed to improve validity of our barriers assessment and provide a more accurate behavioural diagnosis. Indeed, as highlighted in the results, the determinants of behaviour were different depending on the behaviour in question. Thus, if we had only discussed implementing the guidelines as a single behaviour, we may have overlooked key features unique to performing the different behaviours within the guideline. We adhered to the high methodological standards for systematic reviews as recommended by the PRISMA statement and reporting standards outlined by the ENhancing Transparency in REporting the synthesis of Qualitative research (ENTREQ) guidelines [74]. For example, we used an extensive search strategy to identify records, two authors independently screened all titles and full texts for eligibility, and two authors extracted and coded all data, and assessed reporting and methodological rigour of the individual studies. Additionally, backward and forward citations tracking were used to minimise missing studies. Importantly, we have employed the GRADE CERQual approach which allowed us to provide an overall level of confidence to each of our findings.

### Areas for future research

There are several areas for future research in this area. First, while we found 14 studies in this topic area, the majority focused on the behaviour of imaging, the remaining behaviours had fewer studies with contributing data; the lack of data limited our confidence in stating that any of the identified themes reliably explain the behaviour. For example, two other problem behaviours highlighted in the literature, namely overprescribing of opioids and over referral to specialists, had little discussion in the literature. Thus, more research on the barriers to implementing these behaviours is required. Second, while the vast majority of studies focused on the barriers to using the guidelines or performing certain behaviours, we found no reliable evidence on physician-reported facilitators. Thus, future work could include specific questions on assessing facilitators to ensure we get insight into strategies already employed that facilitate the desired behaviour. Third, an important barrier that was identified as a determinant of several behaviours was that of patient demand. For example, physicians reported that a patient’s demand, desire, or wish to have an image be referred to a specialist or have a prescription for a particular medication was often an influencing factor in their decision making. This perception assumes something about the patient and therefore, assessing the patient’s perception is important to correctly address this issue. Several surveys of patients and the general public have found that 50% or more expect diagnostic imaging [75–78]. Two qualitative reviews have investigated patients expectations and experiences of treatment for low back pain, they found a minor theme that patients request imaging in order to get a sick certificate [79], and a major theme that patients perceive medical imaging to offer a definitive diagnosis particularly when they had lost faith in the knowledge of their health professionals [79, 80]. Most interventions that aim to change imaging have targeted the health provider, of those interventions that specifically target patients, most focus on providing pain education or reassurance that prognosis is good, but do not focus on providing a clear diagnosis that will satisfy the patient [81]. Thus, future research could focus on what would be necessary for patients to feel they have received a definitive diagnosis without medical imaging in order to inform patient-targeted interventions. Lastly, we found only one study that included a study interview guide informed by the TDF, which aims to ascertain the physician’s opinion on all domains as either barriers or facilitators, by asking specific questions relating to the 14 TDF domains. Thus, there may be determinants present in other domains than what we found that were missed simply because they were not asked about directly.

## Conclusion

Adopting low back pain guidelines is a well-documented problem, particularly regarding appropriate use of imaging, use of simple analgesics versus opioids for pain relief, and providing advice to stay active. Multiple international campaigns to change these behaviours have been implemented, most with little or no success [82–84]. In this review of the determinants of guideline adherence, we found a high level of confidence in the evidence explaining why physicians find it difficult to adopt two of the five behaviours outlined in the guidelines: (1) image only when needed and (2) refer for adjunct conservative care if patients have not recovered within 4–6 weeks. To reduce unnecessary imaging, it appears we need to target barriers related to social influence, beliefs about consequences, and environmental context and resources. To improve referral to appropriate adjunct conservative care at the right time in the patient’s recovery process, we need to address barriers regarding long wait times or complete lack of access to these services. A number of other barriers were identified in this review. Due to insufficient or low quality supporting data, we were less confident that these could be considered explanatory at this stage. Moreover, healthcare provider behaviour will be influenced to some degree on the healthcare context in which they are practicing. While the barriers we have high confidence in were consistent across multiple health care settings and numerous countries, there are likely additional, context-specific factors that play a role in determining physician behaviour. These factors would have to be considered when designing any intervention.

## Additional files

Additional file 1: Example of search string. (DOCX 12 kb)

Additional file 2: Criteria used for assessing confidence in the evidence supporting the review findings using the CERQual approach. (DOCX 17 kb)

Additional file 3: Assessment of reporting criteria according to the guidance from CASP and COREQ. (DOCX 161 kb)

## Abbreviations

BCT: Behaviour change techniques
CASP: Critical appraisal and skills programme
CERQual: Confidence in the Evidence from Reviews of Qualitative research
COREQ: Consolidated criteria for reporting qualitative research
ENTREQ: ENhancing Transparency in REporting the synthesis of Qualitative research
EQUATOR: Enhancing the QUAlity and Transparency Of health Research
GRADE: Grading of Recommendations Assessment, Development, and Evaluation
LBP: Low back pain
TDF: Theoretical Domains Framework
