# Supplementary material for: Physician-reported barriers to using evidence-based recommendations for low back pain in clinical practice: a systematic review and synthesis of qualitative studies using the Theoretical Domains Framework
Source: Implement Sci. 2019 May 7;14:49. doi: 10.1186/s13012-019-0884-4 (PMC6505266; doi:10.1186/s13012-019-0884-4)
Supplement: Supplementary file 1 — Example of search string. (DOCX 12 kb) [file 13012_2019_884_MOESM1_ESM.docx]

Appendix A

**Example of Search String:**

("intervertebral disk displacement"[tw] OR "Intervertebral Disc Displacement/diagnosis"[Mesh] OR "Intervertebral Disc Displacement/pathology"[Mesh] OR "Intervertebral Disc Displacement/physiology"[Mesh] OR "Intervertebral Disc Displacement/physiopathology"[Mesh] OR "Intervertebral Disc Displacement/psychology"[Mesh] OR "Intervertebral Disc Displacement/rehabilitation"[Mesh] OR "Intervertebral Disc Displacement/therapy"[Mesh] OR "back pain"[tw] OR "Back Pain/diagnosis” OR "Back Pain/pathology" OR "Back Pain/physiology" OR "Back Pain/physiopathology” OR "Back Pain/psychology" OR "Back Pain/rehabilitation" OR "Back Pain/therapy" OR "low back pain"[tw] OR "Low Back Pain/diagnosis"[Mesh] OR "Low Back Pain/pathology"[Mesh] OR "Low Back Pain/physiology"[Mesh] OR "Low Back Pain/physiopathology"[Mesh] OR "Low Back Pain/psychology"[Mesh] OR "Low Back Pain/rehabilitation"[Mesh] OR "Low Back Pain/therapy"[Mesh] OR "pelvic pain"[tw] OR "Pelvic Pain/diagnosis"[Mesh] OR "Pelvic Pain/pathology"[Mesh] OR "Pelvic Pain/physiology"[Mesh] OR "Pelvic Pain/physiopathology"[Mesh] OR "Pelvic Pain/psychology"[Mesh] OR "Pelvic Pain/rehabilitation"[Mesh] OR "Pelvic Pain/therapy"[Mesh] OR "sciatica"[tw] OR "back strain"[tw] OR "non specific back pain"[tw] OR "low back syndrome"[tw] OR "low back dysfunction"[tw] OR "lumbar pain"[tw] OR "backache"[tw] OR "lumbago"[tw]) AND (guideline*[tw] OR "Guideline"[pt] OR "practice guideline"[tw] OR "practice guidelines"[tw] OR "Practice Guidelines as Topic"[Mesh] OR "quality assurance"[tw] OR "Quality Assurance, Health Care"[Mesh] OR "continuing medical education"[tw] OR "Education, Medical, Continuing"[Mesh] OR "decision rules"[tw] OR "decision making"[tw] OR "clinical reasoning"[tw] OR "clinical judgement"[tw] OR "clinical decision"[tw] OR "provider behaviour"[tw] OR "clinician behaviour"[tw] OR "behavioural change"[tw]) AND ("compliance"[tw] OR "adherence"[tw] OR "Guideline Adherence"[Mesh] OR barrier*[tw] OR "resistance"[tw] OR "non compliance"[tw] OR "noncompliance"[tw] OR "Diagnostic Imaging"[Mesh] OR implement*[tw]) AND Humans[Mesh]
