# Supplementary material for: Physician-reported barriers to using evidence-based recommendations for low back pain in clinical practice: a systematic review and synthesis of qualitative studies using the Theoretical Domains Framework
Source: Implement Sci. 2019 May 7;14:49. doi: 10.1186/s13012-019-0884-4 (PMC6505266; doi:10.1186/s13012-019-0884-4)
Supplement: Supplementary file 2 — Criteria used for assessing confidence in the evidence supporting the review findings using the CERQual approach. (DOCX 17 kb) [file 13012_2019_884_MOESM2_ESM.docx]

**Appendix B. Criteria used for assessing confidence in the evidence supporting the review findings using the CERQual approach**

| **Component** | **Definition** | **Threats to component** | **Confidence level not downgraded** | | **Downgrade confidence level** | |
| --- | --- | --- | --- | --- | --- | --- |
|  |  |  | **No or V. minor concerns** | **Minor concerns** | **Moderate concerns** | **Serious concerns** |
| **Methodological limitations** | How much we can trust the findings from the body of data supporting the review finding. This involved identifying whether or not any methodological weaknesses within individual studies impact our confidence. | CERQual suggests assessing issues of recruitment, data collection and analysis; but leaves the criteria selection to review authors.  Upon discussion with our qualitative researcher co-authors and review of with the reporting quality checklists for items related to methodology, we identified 4 areas to assess:   - recruitment methods - data collection methods - assessor influence - data analysis methods   Based on these criteria each individual study’s methodological rigour was determined to be: low, moderate or good.  Threats were considered to be present if the study was assessed to be of Low methodological rigour. | None of the supporting data comes from studies with Low methodological rigour | <25% of the supporting data comes from studies with Low methodological rigour | 25-50% of the supporting data comes from studies with Low methodological rigour | >50% of the supporting data comes from studies with Low methodological rigour |
| **Coherence** | How clear and cogent the fit is between the data from the primary studies and a review finding that synthesizes that data | - Contradictory data - Ambiguous or incomplete data - Competing theories | No threats present in the supporting data | Threats present in <25% of the supporting data | Threats present in 25-50% of the supporting data | Threats present in >50% of the supporting data |
| **Adequacy** | The degree of richness as well as the quantity of data supporting the review finding. | - *Data richness - descriptive findings*: superficial data is ok, - *Data richness - explanatory findings*: superficial data may lack sufficient quality to fully explore the phenomenon - *Data quantity:* one or very few studies or small studies may cause concern. This however, should be taken into context of the review aim and question. If the finding is about a broad phenomenon or large variety of people have less confidence if it is based on small studies. | The supporting data is of sufficient richness and quantity. | The data comes from multiple studies in different settings and varying sample sizes and <25% of the supporting data is too superficial. | The data comes from only a few studies or small studies and 25-50% of the supporting data is too superficial. | The data comes from only a few studies or small studies and >50% of the supporting data is too superficial. |
| **Relevance** | The extent to which the body of data from the primary studies is applicable to the context specified in the review question. | Relevance will be assessed in terms of the following elements of our review question:   - *Population and setting:* Physicians who treat low back pain in primary care settings. - *Phenomenon of interest:* Adopting or performing any of the evidence-based recommendations for managing LBP. - *Outcomes:* barriers or enablers to adopting or performing any of the evidence-based recommendations. | The supporting data is of direct relevance to the review question. | Some of the supporting data (< 25%) is of indirect, partial or unclear relevance. | Some of the supporting data (25-50%) is of indirect, partial or unclear relevance. | The majority of the supporting data (>50%) is of indirect, partial or unclear relevance. |
| Note: Single study rule: for themes with data from a single study only, the following criteria was used to judge methodological limitation: If the study has moderate or low moderate methodological rigour, the confidence level was downgraded. | | | | | | |
