# Supplementary figures and images for: Physician-reported barriers to using evidence-based recommendations for low back pain in clinical practice: a systematic review and synthesis of qualitative studies using the Theoretical Domains Framework
Source: Implement Sci. 2019 May 7;14:49. doi: 10.1186/s13012-019-0884-4 (PMC6505266; doi:10.1186/s13012-019-0884-4)

**Appendix C. Assessment of** reporting criteria according to the guidance from CASP and COREQ.


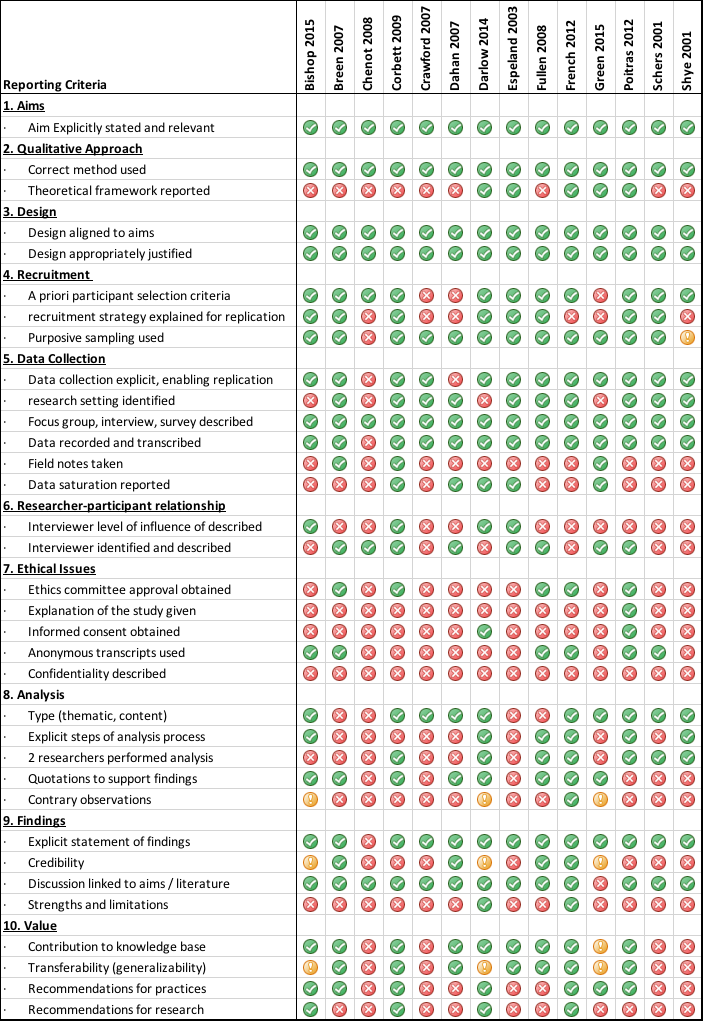

Supplement: Supplementary file 3 — Assessment of reporting criteria according to the guidance from CASP and COREQ. (DOCX 161 kb) [file 13012_2019_884_MOESM3_ESM.docx]
